# Factors associated with stunting among children aged 6–59 months in Bensa District, Sidama Region, South Ethiopia: unmatched case-control study

**DOI:** 10.1186/s12887-021-03029-9

**Published:** 2021-12-06

**Authors:** Temesgen Tafesse, Amanuel Yoseph, Kaleb Mayiso, Taye Gari

**Affiliations:** 1 Department of Public Health, Hawassa University Daye Branch, Hawassa, Ethiopia; 2 School of Public Health, College of Medicine and Health Science, Hawassa University Hawassa, Hawassa, Ethiopia

**Keywords:** Children aged 6–59 months, stunting, unmatched case-control, Bensa district, Ethiopia

## Abstract

**Background:**

Stunting remains one of the most common malnutrition problems among children in Ethiopia. Identifying the risk factors of stunting assists health planners to prioritize prevention strategies, and is a fundamental step for intervention. Therefore, this study aimed to assess factors associated with stunting among children aged 6–59 months in Bensa district, Sidama Region, South Ethiopia, 2018.

**Methods:**

A facility-based unmatched case-control study was conducted from January 10 to March 10, 2018, on a sample of 237(79 cases and 158 controls) children aged 6–59 months with their respective mothers/caretakers. Data were collected using a structured, face-to-face interviewer-administered questionnaire and standard physical measurements. The data were entered into EP INFO version 7 and WHO Anthro software and analyzed using SPSS version 20. The variables were entered into the multivariable model using the backward stepwise regression approach. Multivariable logistic regression analysis was used to identify factors associated with stunting. Adjusted odds ratio (AOR) with 95% confidence interval (95%CI) and p-value <0.05 was used to declare the significance.

**Results:**

Sex distribution was almost equal (Males = 52.3%, Females = 47.7%).The mean (standard deviation) age of cases and controls was 27.35 (±12.71) and 28.70 (±13.27) months respectively.

The risk factors for stunting were diarrhea in the past two weeks (AOR = 2.71, 95% CI: 1.42–5.16), being male (AOR = 2.37, 95% CI: 1.224–4.59), inappropriate exclusive breastfeeding (AOR =2.07, 95%CI: 1.07–4.01), having less than or equal to three under-five children in the household (AOR = 2.18, 95%CI: 1.03–4.64), and mothers who had no formal education (AOR =3.28, 95%CI :1.56–6.924).

**Conclusions:**

Diarrhea in the past two weeks, sex of a child, inappropriate exclusive breastfeeding, number of under-five children in the household, and mothers who had no formal education were the risk factors of stunting. Thus organized efforts aimed at focus on prevention of diarrhea as part of an overall public health strategy for improving child health and nutrition. Educating mothers/caretakers on the importance of exclusive breastfeeding should be considered. Moreover, mothers need to be encouraged to space birth between children through the use of family planning services.

**Supplementary Information:**

The online version contains supplementary material available at 10.1186/s12887-021-03029-9.

## Background

One form of malnutrition is chronic malnutrition or stunting reflects a process of failure to reach linear growth potential. Stunting is defined as a height/length for age is less than −2 SD of the median of the NCHS/WHO international reference. It starts at the beginning of life and lasts throughout their lifetime [1, 2].

Stunting is likely driven by a deficit of essential proteins and micronutrients at the cellular level and chronic inflammation, which reduces the concentration of insulin-like growth factor 1 that is required for linear growth [3]. It is the most prevalent form of malnutrition.

According to the global nutrition report, it was estimated that 150.8 million children had stunted globally in 2017. Regionally, South Asia is home to 38.9% of the world’s stunted children, having the highest burden of the regions. Stunting has declined in Latin America and the Caribbean from 16.9 to 9.6%; and Africa from 38.3 to 30.3% [4]. The burden of stunting is a major public health problem in developing countries (Africa and South Asia) which accounts for two-thirds of the burden [5]. It is also a highly prevalent problem in Ethiopia. According to the Ethiopian DHS 2016 report, 38% of under-five children were stunted. Similarly, the high prevalence was reported in South Ethiopia 38.6% [6]. A study conducted in the region showed that the prevalence of child stunting was 41% [7].

Malnutrition is responsible for more than one-third of child deaths and 11% of the global total disease burden [8]. The economic cost of stunting is high which leads total loss of 15% disability-adjusted life years [9]. It has also resulted in a loss of adult height by 1%. Consequently, childhood stunting might end up with 1.4% losses in productivity [10].

Health consequences of stunting are delay in physical growth and motor development, and susceptibility to contracting diseases. Later in life, it increases the risk of being overweight and developing associated chronic diseases such as cardiovascular disease, diabetes, cancer, and mental health disorders [11, 12]**.**

Malnutrition is the result of a long sequence of interlinked events. The most frequently suggested causes of malnutrition are: Immediate cause, underlying cause, and basic cause. The immediate causes are poor diet and disease which are themselves caused by underlying determinants; household food security, maternal/child-caring practices, and access to health services, and a healthy environment. Underlying factors themselves are influenced by the basic causes; socio-economic and political conditions [13]. Thus, two main pathways are described leading to a child’s growth failure: inadequate nutrition and exposure to infection/inflammation [14].

Sustainable Development Goals (SDGs) contain indicators that are highly relevant for nutrition, reflecting nutrition is a central role in sustainable development. Global nutrition target for 2025 to reduce the number of stunted children by 40% [15].

Ethiopia had established rural development extension strategies and related programs to address the malnutrition problem. The outcomes of initiatives are encouraging but, not sufficient due to a lack of coordination among the relevant sectors. In addition, Ethiopia launched an ambitious and revised National Nutrition Plan (NNP) primarily focused on the first 1000 days and accelerated stunting reduction actions. It was also targeted to reduce the prevalence of stunting from 44.4 to 20% [16]. Thus, the Food and Nutrition Policy (FNP) was developed with the involvement of relevant stakeholders to address the food and nutrition security challenges of the country through multi-sectoral integration and collaboration. It aims to end child stunting in Ethiopia [17]. However, under-nutrition remains one of the child health problems and poses a significant obstacle to achieving better child health outcomes [18].

There is limited information on factors associated with stunting among children aged 6–59 months in Bensa district, Sidama Region, South Ethiopia. In addition, information is also important because of the target for Second Growth and Transformation Plan (GTPII) in 2020 [19]. And also the preparation for the third Growth and transformation plan in Ethiopia is fast approaching and the findings from this study serve as baseline information. Moreover, assessing and identifying the risk factors for stunting is important to guide public health planners, policymakers, and implementers to plan and design appropriate intervention strategies to enhance the nutritional status of the children. Therefore, the main aim of this study is to assess factors associated with stunting among children aged 6–59 months in Bensa district, Sidama Region, South Ethiopia.

## Methods

### Study setting, design, and population

A facility-based unmatched case-control study was carried out in Bensa district from January 10 to March 10, 2018. Bensa district is located 400 km from Addis Ababa, the capital of Ethiopia. It is also 131 km from Hawassa, the capital of Sidama Region. According to the 2018 central statistical agency report of Ethiopia, the total population of the district was estimated to be 310, 952 (8.1% urban and 91.9% rural). Of these, 13.94% were children in the age group of 6–59 months. The district has consisted of 03 urban and 34 rural Kebeles (smallest administrative unit of Ethiopia). The health service coverage of the district was 94%. There are a one-government primary hospital, 11 health centers, and 37 health posts. It has also consisted of 4 private clinics and 12 pharmacies in the districts. According to the health department report, the distribution of stunting affects almost all Kebeles of the district.

The source population of this study was all children in the age group of 6–59 months (pair with their mothers/caretakers) who were utilized EPI (Expanded Program for Immunization) and under-five OPD (Out Patient Department) service in Bensa district health facilities. The sample population was all selected children in the age group of 6–59 months (pair with their mothers/caretakers) in selected health facilities who have lived with the child at least for 6 months. Those children who were very sick requiring emergency treatment were excluded from this study.

### Sample size determination and sampling technique

The sample size was calculated by using a two population proportion formula in consideration of the following assumptions: the proportion of children who were sick every month be 14.6% for controls and of the cases 32.4%, level of confidence 95%, power of the study 80%, a ratio of controls over cases 2:1 to detect an odds ratio of 2.8 [20]. Thus, the final sample size after adding a 10% non-response rate is 237 (79 cases and 158 controls). In Bensa district, there is 1 primary hospital and 11 health centers. One primary Hospital and three Health centers were selected out of eleven health centers by simple random sampling technique (lottery method). The calculated sample size (237) was proportionally allocated to the selected health facilities. A consecutive sampling technique was used to select the study participants until the calculated sample size was attained. All children aged 6 to 59 months visiting hospital and health centers during the data collection period were measured for their height. Then, children were categorized as stunted or non-stunted based on the calculated z-score value. First, stunted children were identified and then selected as cases. The controls were children aged 6 to 59 months without stunting from the same facility cases where selected.

### Data collection method and tools

The data collection was administered by 9 Bsc nurses. One health officer and the principal investigator intensively supervised the data collection process. The data collectors were collected information related to factors associated with stunting by interviewing the mothers/caretakers face to face. A structured questionnaire and anthropometric measurement were used to collect data. Interviews were conducted with mothers/caretakers of the children to fill the questionnaire and were impossible to get them or if there is refusal, the next client was considered for the study. Data collection was conducted in a stepwise manner in each health facility in their respective schedule.

### Anthropometric measurements

The body length of children aged 6–24 months was measured without shoes and the length will read to the nearest 0.1 cm. The mother helped to measure the recumbent length of her child and this was done by using a portable horizontal wooden length board. However, the height of children aged 24–59 months was measured using a vertical wooden height board by placing the child on the measuring board, and the child standing upright in the middle of the board. The child’s head, shoulders, buttocks, knees, and heels touch the board.

### Data quality control

Data were collected using a structured, face-to-face interviewer-administered questionnaire and standard physical measurements. To ensure the quality of data pre-tested was done on 5% of samples in the non-selected health facility. A pre-tested questionnaire was prepared in English and translated into the local Sidamigna language and retranslated back to English to ensure consistency. The training was given to data collectors and supervisor by the principal investigator for two days. The training was focused on the objectives of the study and data collection method, anthropometric measurement, and data recording. Regular checkups for completeness and consistency of the data were made daily.

### Study variables

The outcome variable (Height/length for age z-score, Stunting).

Independent variables included Socio-demographic variables(marital status, residence, ethnicity, religion, number of under-five children, family size, parent’s education status, occupation, and economic status), Child characteristics (age, sex, birth order, birth interval, place of delivery, types of birth, and morbidly status), Child caring practices (feeding and immunization), maternal characteristics(age, mothers’ age during first child, number of children ever have born, ANC visits, use of extra food during pregnancy or lactation and family planning), environmental Health condition (water, hygiene, and sanitation).

### Operational definitions

Stunting (chronic malnutrition): means HFA is below −2 SD of the reference population while below −3 SD indicates severe stunting. Controls: were defined as study subjects who had an anthropometric reading with z-scores ≥ − 2SD [2].

Duration of breastfeeding: the number of months of breastfeeding among children.

Complementary foods: are foods which are required by the child after six months of age.

Diarrhea: a child with loose stools three or more times in a day.

Family size: refers to the total number of people living in a house during the study period.

### Data processing and analysis

The data were entered into EP INFO version 7 and WHO Anthro software and analyzed using SPSS version 20. The wealth index was constructed by using Principal Components Analysis in SPSS. All required variables recording and computations were done before the main analysis. Descriptive analyses such as mean, standard deviation, proportion were conducted to obtain descriptive measures for the socio-demographic characteristics and other variables.

Binary logistic regression was used to identify factors associated with stunting. The bi-variable logistic regression analysis started with unadjusted analysis in which each potential predictor was assessed separately for its association with stunting [21]. Variables with p-values <0.25 on the unadjusted analysis were entered into a multivariable logistic regression model to find out independent risk factors of stunting adjusting for other factors in the model.

Moreover, multicollinearity was checked by using the collinearity diagnostic test by checking the value of the Variance Inflation Factor (VIF) and tolerance test. In this study, the maximum VIF became 4.71 while the tolerance value was greater than 0.1, which proved the absence of multicollinearity between the independent variables. Hosmer–Lemeshow goodness-of-fit was used to test for the model fitness, and the p-value for the Hosmer-Lemeshow test was 0.49 which indicates a good model.

Odds ratio at 95% CI were used to measure the strength of association between outcome and predictor variables. *P*-value <0.05 was considered to declare statistical significance in multivariate logistic regression analysis. Finally, the results were presented in texts and tables.

## Results

### Socio-economic and demographic characteristics of the parents

A total of 237 participants have participated in the study with a response rate of 100%. The majority of study subjects were Sidama in both groups; 75 (94.9%) of cases and 154 (97.5%) of controls. Similarly, 74 (93.7%) mothers of children in cases and 151 (95.6%) in controls were protestant in religion. Mothers of 45 (57.0%) of cases and 48 (30.4%) controls had no formal education. Households who had less than or equal to three under-five children were 40 (50.6%) and 47 (29.7%) of cases and controls respectively. Similarly, the family size was five and above for 46 (58.2%) of cases and 70 (44.3%) of controls (Table 1).

**Table 1:** Socio-economic and demographic characteristics of study participants in Bensa district, Sidama Region, South Ethiopia, 2018

Variables Category		Cases(***n*** = 79)		Controls(***n*** = 158)		***P***-value
		Frequency	%	Frequency	%	
Religion	Protestant	74	93.7	151	95.6	.530
	Others^a^	5	6.3	7	4.4	
Ethnicity	Sidama	75	94.9	154	97.5	.309
	Others^b^	4	5.1	4	2.5	
Residence	Rural	66	83.5	120	75.9	.180
	Urban	13	16.5	38	24.1	
Household head	Father	52	65.5	72	45.6	.360
	Mother	27	34.2	86	54.4	
Marital status	Married	73	92.4	150	94.9	.436
	Others^c^	6	7.0	8	5.1	
Family size	< 5	33	41.8	88	55.7	.043
	≥5	46	58.2	70	44.3	
Number of <5 yr children	1	39	49.4	111	70.3	.002
	2–3	40	50.6	47	29.7	
Educational status of mother	Has no formal education	45	57.0	48	30.4	.001
	Can read and write	9	11.4	11	7.0	
	Primary (1–8)	13	16.4	72	45.6	
	Secondary & above	12	15.2	27	17.1	
Educational status of father	Has no formal education	14	17.7	18	11.4	.130
	Can read and write	12	15.2	15	9.5	
	Primary(1–8)	34	43.0	67	42.4	
	Secondary & above	19	24.0	58	36.7	
Mother’s occupation	Housewives	66	83.5	134	84.8	.800
	Others^d^	13	16.5	24	15.2	
Father’s occupation	Farmers	57	72.2	96	60.8	.084
	Others^d^	22	27.8	62	39.2	
Economic status of families	Poor	41	51.9	53	33.5	.008
	Medium	17	21.5	32	20.3	
	Rich	21	26.6	73	46.2	

Key: ^a^ = Muslim, Orthodox, Catholic; ^b^ = Oromo, Amhara, Gurage, Wolayta; ^c^ = single, divorced, widowed;^d^ = merchant/Trade, private org. employee, daily laborer, a government employee

### Child characteristics

The mean age of cases and controls was 27.35 (±12.71) and 28.70 (±13.27) months respectively. The largest proportion of cases (30.4%) and controls (27.8%) of the children were found in 12–23 months age groups. Among the cases, 65.8% were male and 45.6% were females. More than half (54.4%) of cases and 67.1% controls were delivered at the health facility. Among the study participants, 44 (55.7%) of cases and 51 (32.3%) of controls had diarrhea in the past 2 weeks before the survey (Table 2).

**Table 2:** Child characteristics among children aged 6–59 months in Bensa district, Sidama Region, South Ethiopia, 2018

Variables	Category	Cases (***n*** = 79)		Controls (***n*** = 158)		***P***-value
		Frequency	%	Frequency	%	
Sex of children	Male	52	65.8	72	45.6	.003
	Female	27	34.2	86	54.4	
Age in months	6–11	7	8.9	16	10.1	.993
	12–23	24	30.4	44	27.8	
	24–35	20	25.3	40	25.3	
	36–47	19	24.1	40	25.3	
	48–59	9	11.4	18	11.4	
Birth order	1	19	24.1	61	38.6	.003
	2–3	27	34.2	63	39.9	
	***≥***4	33	41.8	34	21.5	
Place of delivery	Home	36	45.6	52	32.9	.057
	Health facility	43	54.4	106	67.1	
Home delivery by	TBA	6	16.7	7	13.5	.677
	Neighbors	30	83.3	45	86.5	
Birth type	Single	78	98.7	155	98.1	.721
	Multiple	1	1.3	3	1.9	
Birth interval	<2 year	19	24.1	25	15.8	.013
	***≥***2	47	59.5	79	50.0	
	No previous birth	13	16.5	54	34.2	
Diarrhea, last 2wks	No	35	44.3	107	67.7	.001
	Yes	44	55.7	51	32.3	
Fever, last 2wks	No	47	59.5	113	71.5	.062
	Yes	32	40.5	45	28.5	
ARI, last 2wks	No	72	91.1	134	84.8	.173
	Yes	7	8.9	24	15.2	

### Child care practices

Of all mothers of children in the cases group, 50 (63.3%) and in the controls group, 118(74.7%) started breastfeeding within the first one hour after birth. The exclusive breastfeeding rate for 6 months were 34 (43.0%) and 106 (67.1%) for cases and controls respectively. Meanwhile, the duration of breastfeeding was less than 2 years for 46 (58.2%) and 86 (54.4%) of cases and controls respectively. Concerning immunization status 67 (84.4%) of cases and 146 (92.4%) of controls were vaccinated, and 35(44.3%) of cases and 90(57.0%) of controls were received deworming services (Table 3).

**Table 3:** Child caring practices of mothers/caretakers of children aged 6–59 months in Bensa district, Sidama Region, South Ethiopia, 2018

Variables	Category	Cases(***n*** = 79)		Controls(***n*** = 158)		***P***-value
		Frequency	%	Frequency	%	
Breast milk initiated after delivery	Immediately	50	63.3	118	74.7	.069
	After 1 to 24 h	29	36.7	40	25.3	
Pre-lactation food/fluid	No	69	87.3	136	86.1	.788
	Yes	10	12.7	22	13.9	
Type of pre-lactation food/fluid	Water	5	50	10	45.5	.811
	Others^a^	5	50	12	54.5	
Duration of EBF	<6 or > 6 months	45	57.0	52	32.9	.001
	6 months	34	43.0	106	67.1	
Complimentary Feeding	<6 or > 6 months	45	57.0	52	32.9	.001
	6 months	34	43.0	106	67.1	
Frequency of feeding/day	2 times	13	16.5	20	12.7	.607
	3 times	36	45.6	69	43.7	
	≥4 times	30	38.0	69	43.7	
Method used for feeding	Bottle	21	26.6	28	17.7	.274
	Hand	19	24.1	40	25.3	
	Spoon and Cup	39	49.4	90	57.0	
Duration of B/F	< 24 months	46	58.2	86	54.4	.579
	≥24 months	33	41.8	72	45.6	
Immunization	No	12	15.2	12	7.6	.068
	Yes	67	84.4	146	92.4	
VAS	No	15	19.0	17	10.8	.081
	Yes	64	81.0	141	89.2	
Deworming	No	44	55.7	68	43.0	.066
	Yes	35	44.3	90	57.0	

Key: ^a^ = Milk, Butter, Hamesa

### Maternal characteristics

The mean age for mothers of the cases was 29.25 (±5.88) while it was 27.30 (±5.17) for the mothers of the controls. Mothers of 33 (41.8%) of cases and 40 (25.3%) of controls had given birth of 4 and above children. Mothers of 52 (65.8%) of cases and 82 (51.9%) of controls didn’t consume extra food during pregnancy and lactation. About 62 (78.5%) and 132 (83.5%) of mothers visited health facilities for antenatal care during their pregnancy period for both cases and controls respectively. Concerning family planning, mothers of 61 (77.2%) of cases and 125 (79.1%) of controls were every used family planning (Table 4).

**Table 4:** Maternal characteristic of children aged 6–59 months in Bensa district, Sidama Region, South Ethiopia, 2018

Variables		Cases (***n*** = 79)		Controls (***n*** = 158)		***P***-value
	Category	Frequency	%	Frequency	%	
Mothers age	<20 years	7	8.9	17	10.8	.127
	20–35 years	60	75.9	130	82.3	
	>35 years	12	15.2	11	7.0	
Mothers age during first child	<21 years	60	75.9	110	69.6	.308
	***≥***21 years	19	24.1	48	30.4	
Number of children	<4	46	58.2	118	74.7	.010
	***≥***4	33	41.8	40	25.3	
Extra food during pregnancy and lactation	No	52	65.8	82	51.9	.041
	Yes	27	34.2	76	48.1	
ANC for this child	No	17	21.5	26	16.5	.340
	Yes	62	78.5	132	83.5	
F/P every used	No	18	22.8	33	20.9	.737
	Yes	61	77.2	125	79.1	

### WASH condition of study participants

Children of households who used unprotected sources of water for drinking accounts 32 (40.5%) and 43 (27.2%) in cases and controls respectively. Those children households who didn’t treat water by any means were found to be 61 (77.2%) in cases and 113 (71.5%) in controls. The majority of children of HHs, 73 (92.4%) of cases, and 148 (93.7%) of controls have a latrine. (Table 5).

**Table 5:** WASH conditions of mothers/caretakers of children aged 6–59 months in Bensa district, Sidama Region, South Ethiopia, 2018

Variables	Category	Cases(***n*** = 79)		Controls(***n*** = 158)		***P***-value
		Frequency	%	Frequency	%	
Drinking water source	Unprotected^a^	32	40.5	43	27.2	.038
	Protected^b^	47	59.5	115	72.8	
Treat water by any means	No	61	77.2	113	71.5	. 349
	Yes	18	22.8	45	28.5	
Having latrine	No	6	7.6	10	6.3	.714
	Yes	73	92.4	148	93.7	
Wash hand after toilet	No	15	19.0	39	24.7	.324
	Yes	64	81.0	119	75.3	
The material used to wash hands after toilet	Using water only	34	53.1	55	46.2	.096
	Water and soap	20	31.2	54	45.4	
	Water and ashe	10	15.6	10	8.4	
Flush toilet	No	58	73.4	96	60.8	.054
	Yes	21	26.6	62	39.2	
The method used for disposal of HHs waste	Open field	16	20.3	18	11.4	.113
	In-pit	16	20.3	46	46	
	Composting	41	51.9	74	74	
	Burning	6	7.6	20	20	

Key: ^a^ = river, pond, unprotected well; ^b^ = public tap, private tape, protected well**.**

### Factors associated with stunting among children aged 6–59 months

In the bivariate analysis, the number of under-five year’s children in the households, sex of a child, mothers’ educational status, economic status of a family, total family size, diarrhea last 2wks, inappropriate exclusive breastfeeding, and source of drinking water had a significant association with stunting.

The multivariable model rivaled that children living in households less than or equal to three under-five children were 2.18 times more likely to develop stunting as compared to those living in households with one under-five child (AOR = 2.18, 95% CI :1.03–4.64). The odds of stunting in male children were 2.4 times higher as compared to females (AOR = 2.37, 95% :1.224–4.59).

This model also showed that children whose mothers have no formal education were 3.28 times more likely to be stunted as compared to mothers with formal education (AOR = 3.28, 95%CI:1.56–6.924). Children who inappropriately exclusively breastfeed were 2.07 times more likely to develop stunting than children who were exclusively breastfed for the first 6 months (AOR = 2.07, 95% CI: 1.07–4.01). Children with exposure to diarrhea last 2 weeks were 2.71 times more likely to be stunted when compared with those children with no diarrheal exposure (AOR = 2.71, 95%CI :1.42–5.16) (Table 6).

**Table 6:** Bi-variable and multivariable logistic regression analysis of the factors associated with stunting among children aged 6–59 months in the Bensa district, Sidama Region, South Ethiopia, 2018

Variables	Cases	Controls	COR(95%CI)	AOR(95%CI)
Sex of children				
Male	52	72	2.300 (1.313–4.029)**	2.37 (1.224–4.59)*
Female	27	86	1	1
Total family size				
***≥***5	46	70	1.752 (1.015–3.036)*	.492(.174–1.389)
<5	33	88	1	1
Number of <5 year children				
2–3	40	47	2.422 (1.387–4.230)**	2.18 (1.03–4.64)*
1	39	111	1	1
Maternal educational status				
Has no formal education	45	48	3.712 (2.041–6.753)***	3.28 (1.56–6.924)**
Can read & write	9	11	3.240 (1.211–8.669)*	2.752(.897–8.446)
Has formal education	25	99	1	1
Wealth Index				
Poor	41	53	2.689 (1.427–5.068)**	1.169(.538–2.539)
Medium	17	32	1.847(.861–3.959)	.906(.360–2.281)
Rich	21	73	1	1
Diarrhea, preceding 2wks				
Yes	44	51	2.638 (1.514–4.596)**	2.71 (1.42–5.162)**
No	35	107	1	1
Duration of EBF				
< 6 or > 6 months	45	52	2.698 (1.548–4.702)	2.07 (1.06–4.01)*
6 months	34	106	1	1
Source of drinking water				
Unprotected	32	43	1.821 (1.030–3.219)	1.446(.738–2.833)
Protected	47	115	1	1

Key: 1: Reference category; * = *P* < .05; **: *P* < .005;***P < .001; *COR* Crude Odds Ratio**,** *AOR* Adjusted Odds Ratio**,** *CI* Confidence Interval

## Discussion

In this study factors associated with stunting were having less than or equal to three under-five children in the household, male sex, mothers who had no formal education, inappropriate exclusive breastfeeding, and exposure to diarrheal diseases in the last 2 weeks preceding the survey.

In this study participants living in households with less than or equal to three under-five children are more likely to develop stunting as compared to those living in households with one under-five child. This finding is similar with the studies conducted in Gurage Zone, Mekelle City, and Mozambique [22–24]. This might be the fact that as the number of under-five children increases in the household; the care given to the children decreases, mothers are unable to optimally breastfeed children, causes competition on family resources, and increases the risk of infectious diseases [25].

From this study finding, the odds of stunting are significantly higher in male children than females. This result is consistent with studies done in Lasta district, Korahay Zone, Dollo ado district, multi-level analysis in Ethiopia, Southwest Uganda, and Indonesia [26–31]. The cause of this difference in sex requires further study, but variation might be due to the sex preference of the family. Additionally, it is believed that boys to be biologically more vulnerable to morbidity [32]. In contrast, a study conducted in Merhabete district, and Pakistani showed that female children were more stunted compared to male children [33, 34]. But this discrepancy could not be important to design interventions; because sex is a non-modifiable risk factor.

Children whose mothers had no formal education were more likely to be stunted as compared to have a formal education. This study is in line with studies conducted in Hossana Town, rural Uganda, Iran, Kenya, and Padang city [35–39]. Mother’s education affects the knowledge and attitude of parents which in turn affect their fertility behavior, use of health services, and access to nutrition-related information. And also maternal education influences the preparation, procurement, and selection of nutritious foods for themselves and their children. The cumulative effect of these can have an impact on the prevention of stunting [40].

Appropriate infant and young child feeding has been identified as one of the key determinants of child malnutrition particularly stunting [41]. In the current study, inappropriate exclusive breastfeeding children were more likely to be stunted compared to their counterparts. This result is agreed with studies conducted in rural Dubit district, Arba Minch, and Nepal [42–44]. This might be early initiation and exclusive breastfeeding for 6 months of life meets the energy and nutrient needs of the vast majority of infants and provides protection against gastrointestinal infections, which can lead to severe nutrient depletion and stunting [45]. Furthermore, after 6 months breast milk alone is not sufficient to meet the nutritional requirements. Both earlier and delayed introduction of complementary food predisposes the child to increased risks of growth faltering [46].

This study also showed that children with diarrhea exposure in the past two weeks were more likely to be stunted compared to those children with no exposure. This finding is in line with the finding of studies conducted in Kindo Didaye district, Bule Hora district, Angolela Tera District, and Kenya [47–50]. The possible explanation might be increased exposure to infections associated with taking other fluids, solids, and ingestion of contaminated materials as the children start exploring their environment. Diarrhea affects children’s nutritional status by diminishing appetite, reducing nutrient absorption, increasing metabolic requirements, and increasing nutrient losses [51].

### Limitation of the study

This study had several strengths. Among these, many variables considered to be risk factors of stunting were assessed and a pre-tested and valid questionnaire was used to collect information. The case-control nature of this study is stronger than a cross-sectional study in assessing risk factors which is important to develop a relevant policy strategy for efficient prevention of stunting. Regardless of its strengths, our study had some basic limitations that might be considered while interpreting the results. First, the study might be prone to recall bias because the study was based on a self-report of questions that require a good memory of mother’s/care giver. Second, the study is predisposed to errors of anthropometric measurement that might lead to misclassification of children’s nutritional status. Third, the WASH variables were not significantly associated with stunting. This is due to there is a possibility of reporting bias both from respondents and data collectors. Cognizant of this, data collectors were told during the training to properly ask WASH variables and to elicit a genuine response from the respondents. Fourth, similar to several other observational studies, we controlled for potential confounders using the multivariable logistic regression model. However, we were not considered all possible risk factors of stunting due to the resource and time shortage. Moreover, we did not collect data on food security, minimum dietary diversity score, and the occurrence of intestinal helminths on infections that could contribute to the increased prevalence of malnutrition.

## Conclusions

The number of under-five year old children in the household, sex of a child, mother’s educational status, inappropriate exclusive breastfeeding, and diarrhea in the past 2 weeks were significant associated factors with stunting.

This study finding has serious policy implications because it provides a basis for expanding stunting reduction interventions in rural areas to include not only nutritional but also personal hygiene and environmental sanitation measures. The appropriate infant and young child feeding, maternal education, birth spacing, and healthier environment of the child with reduced exposure to bacteria have a substantial potential to further reduce stunting.

Therefore, the Bensa district health office should encourage mothers to space birth between children through the use of family planning services. Health extension workers shall educate mothers/caretakers on the importance of exclusive breastfeeding. Moreover, the district health office should give priority to personal hygiene and environmental sanitation to prevent diarrhea.

## Supplementary Information

**Additional file 1. S1 Tool.** This is the S1 English version survey questionnaire.

## Data Availability

The datasets used and/or analyzed during the current study available from the corresponding author on reasonable request.

## Abbreviations

AOR: Adjusted odds ratio
CI: Confidence Interval
EPI: Expanded program for Immunization
GTPII: Growth and transformation plan two
OPD: Out Patient Department
SD: Standard Deviation
SDG: Sustainable Development Goals
OR: Odd ratio
SPSS: Statistical packages for social science
WASH: Water, sanitation and hygiene
WHO: World Health Organization
