# Supplementary material for: Factors associated with stunting among children aged 6–59 months in Bensa District, Sidama Region, South Ethiopia: unmatched case-control study
Source: BMC Pediatr. 2021 Dec 6;21:551. doi: 10.1186/s12887-021-03029-9 (PMC8647487; doi:10.1186/s12887-021-03029-9)
Supplement: Supplementary file 1 — Additional file 1. S1 Tool. This is the S1 English version survey questionnaire. [file 12887_2021_3029_MOESM1_ESM.docx]

## Annex 1 ፡ English Version Questionnaires

Hawassa University College of Medicine and Health Sciences, School of Public and Environment Health. Questionnaires developed to assess risk factors associated with stunting among children aged 6-59months in Bensa district , Sidama Region, South Ethiopia ; 2018.

001. MRN ID number፡-----------------------

002. Address; Kebele ፡ ----------------------Got------------------House number----------------------

| PART ONE: SOCIO-DEMOGRAPHIC INFORMATION | | | |
| --- | --- | --- | --- |
| No | Questions | Responses | Remark |
| 101 | What is the sex of household head? | 1.Male  2.Female |  |
| 102 | Ethnicity? | 1. Sidama 3. Oromo  2.Amhara 4.Gurage  5.Wolayta 6.Other(specify)------ |  |
| 103 | \What is your religion? | 1.Protestant 3.Muslim  2. Orthodox 4. Catholic  5. Others(specify) |  |
| 104 | Marital status? | 1. Married 3.Divorced  2. Widowed 4. Single |  |
| 105 | Total family size (How many person live in the HH) | In number_____ |  |
| 106 | How many children <5 year live in the HH? | In number_____ |  |
| 107 | Maternal level of education? | 1. Has no formal education  2. Read and write  3. Formal education |  |
| 108 | If formal education, specify the highest grade attained? | __________ |  |
| 109 | Paternal level of education? | 1. Can't read and write  2. Read and write  3. Formal education |  |
| 110 | If formal education, specify the highest grade attained? | __________ |  |
| 111 | Occupation of mother ? | 1. House wife  2. Farmer  3. Merchant/Trade  4 Private Organization employee  5. Government employee  6. Daily laborer  7. Other (specify) |  |
| 112 | Occupation of father? | 1.Farmer  2.Government employee  3.Merchant/Trade  4. Private Org. employee  5.Daily laborer  6. Other (specify) |  |
| \| PART TWO ፡ ECONOMIC INFORMATION \| \| \| \|  \| \| --- \| --- \| --- \| --- \| --- \| \| No \| Questions \| Re ponses \| Remark \| \| 201 \| Does your household own any land that can be use  for agriculture? \| 1.Yes  2.No \| If no, skip to 206 \| \| 203 \| How many (local units) of agricultural  land does the household own? Ask them how many oxen)  (4oxens = 1 hectare= 1000^m2^) \| 1. ____________ Units. Write the local unit here  2. I don’t know/Not sure \|  \| \| 204 \| Does this household own any livestock, herds, or farm animals? \| 1.Yes  2..No \|  \| \| 205 \| How many of the following animals does this household own?  Milk cows or oxen?  Horses, donkeys, or mules?  Goats or sheep?  Chickens? \| --------------------  --------------------  --------------------  -------------------- \|  \| \| 206 \| Does your household have:  Electricity?  A watch?  A radio?  A television?  A mobile telephone?  A non-mobile telephone?  A refrigerator?  A table?  A chair?  A bed? \| Yes No  Electricity………….…………..1 2  Watch………………………….1 2  Radio……………………..…....1 2  Television……………………....1 2  Mobile Telephone…………………1 2  Non-Mobile Telephone………..…...1 2 Refrigerator……………………1 2  Table…………………..……….1 2  Chair……………………………1 2  Bed…………………………..…1 2 \|  \| \| 207 \| Does any member of this household own:  A bicycle?  A motorcycle?  An animal-drawn cart?  A car or truck? \| Yes No  Bicycle……….………………..1 2  Motorcycle……………………..1 2  Animal-drawn cart……………...1 2  Car/truck……………...………..1 2 \|  \| \| 208 \| What is the main material of the floor of your house? \| 1. Natural floor  2. Wood or bamboo floor  3. Finished floor  4. If other specify __________ \|  \| \| 209 \| What is the main material of the roof your house? \| 1. Thatched/Leaf  2. Plastic sheet  3. Corrugated iron  4. If other specify __________ \|  \| \| 210 \| What is the main material of the wall of your house? \| 1. Bamboo/Wood  2. Stone with mud  3. Trunks with mud  4. Bricks/Cement  5. If other specify __________ \|  \| \| 211 \| Does any member of this household have an account with a bank/credit  association/ microfinance? \| 1. Yes  2. No  3. I don’t know/Not sure \|  \| \| 212 \| What type of fuel does your household  mainly use for cooking? \| 1. Wood  2. Charcoal  3. Animal dung  4. Kerosene  5. If other specify __________ \|  \| | | | |

| PART THREE: CHILD CHARACTERSTICS | | | | | |
| --- | --- | --- | --- | --- | --- |
| No | | Questions | | Responses | Remark |
| 301 | | Child's sex? | | 1. Male  2. Female |  |
| 302 | | Child’s age? | | _____ Months |  |
| 303 | | Birth order? | | ____ ^th^ |  |
| 304 | | Place of delivery? | | 1.Home  2.Health facility |  |
| 305 | | If place of delivery in the home, specify? | | 1.TBA  2.Neighbours  3.Others (specify) |  |
|  | |  | |  |  |
| 306 | | Type of birth? | | 1. Single  2. Multiple/Twin/ |  |
| 307 | | Birth interval b/n the youngest and his/her immediate elder in years? | | 1.<2 years  2.2years  3.>2years  4. No previous birth |  |
| 308 | | Had the child had diarrhea in  the last two Weeks? | | 1.Yes  2. No |  |
| 309 | | Had the child had fever last two weeks? | | 1. Yes  2. No |  |
| 310 | | Had the child had acute respiratory disease in the last two weeks? | | 1. Yes  2. No |  |
| 311 | | Height of a child | | _______cm |  |
|  | | | | | |
| PART FOUR: CHILD CARING PRACTICE | | | | | |
| No | Questions | | Responses | | Remark |
| 401 | When did you start to breast feed the child after birth? | | 1. Immediately  2. After 1 to 24 hrs  3. If other(Specify) | |  |
| 402 | Did you give the child pre-lactation Food/fluid? | | 1. Yes  2. No | |  |
|  |  | |  | |  |
| 403 | If yes, what did you gave him (her)? | | 1. Water 3. Milk  2. Butter 4.Hamesa  5..Other(specify) | |  |
| 404 | How long did you exclusively breast feed this child? | | Months | |  |
| 405 | When did you start complementary feeding?(Age in months) | | ____________Months | |  |
|  |  | |  | |  |
| 406 | How many times the child’s feeding per day? | | 1.One times 3.Three times  2. Two times 4. Three times and above | |  |
| 407 | What kind of method used for feeding? | | 1. Bottle 3. Hand  2. Cup 4, Spoon | |  |
| 408 | Duration of breast feeding? | | 1.<1 year  2.1-2 years  3.≥2 years | |  |
| 409 | Was the child immunized for his age?(check BCG scar) | | 1. Yes  2. No | |  |
| 410 | Vitamin A supplementation in the past six months? | | 1. Yes  2. No | |  |
| 411 | Did a child receive de-worming last six month? | | 1.Yes  2.No | |  |

| PART FIVE : MATERNAL CHARACTERSTICS | | | | |
| --- | --- | --- | --- | --- |
| No | Questions | | Responses | Remark |
| 501 | Mother’s age in years? | | ______ year completed |  |
| 502 | Age at first birth (year)? | | _____ years |  |
| 503 | Total number of children ever born? | In number _______ | |  |
| 504 | During pregnancy or lactation, did you consume extra food? | 1. Yes  2. No | |  |
| 505 | Did you visit health facility for ANC? | 1. Yes  2. No | |  |
|  |  | |  |  |
| 506 | Do you use family planning? | | 1. Yes  2. No |  |
| \| **PART SIX: WATER, SANITATION AND HYGIENE** \| \| \| \| \| \| \| --- \| --- \| --- \| --- \| --- \| --- \| \| No \| Questions \| \| Responses \| Remark \| \| \| 601 \| What is your main source of drinking water? \| \| 1. River 3.Protected well  2. Pond 4. Unprotected well  5.Public tap 6.Private tap \|  \| \| \| 602 \| Do you treat water by any means? \| \| 1.Yes  2. No \|  \| \| \| 603 \| Do you have latrine? \| \| 1. Yes  2. No \|  \| \| \| 604 \| Do you wash hand after toilet? \| \| 1.Yes  2.No \|  \| \| \| 605 \| What kind of material used to wash hands after toilet? \| \| 1.Using water only  2. Using water and Soaps  3.Using water and Ashe \|  \|  \| \| 606 \| Do you use water to clean  Toilet? \| \| 1.Yes  2.No \|  \|  \| \|  \| \|  \|  \|  \|  \| \| 607 \| What kind of method used for disposal of HHs waste? \| \| 1. Open field  2. In a pit  3. Common pit  4. Composting  5. Burning \|  \| \|   Name of interviewer--------------------------------Signature------------------Date of interview------    **THANK YOU FOR YOUR COOPERATION!** | | | | |
